# Evaluation of Study and Patient Characteristics of Clinical Studies in Primary Progressive Multiple Sclerosis: A Systematic Review

**DOI:** 10.1371/journal.pone.0138243

**Published:** 2015-09-22

**Authors:** T. Ziemssen, S. Rauer, C. Stadelmann, T. Henze, J. Koehler, I.-K. Penner, M. Lang, D. Poehlau, M. Baier-Ebert, H. Schieb, S. Meuth

**Affiliations:** 1 University Clinic Carl Gustav Carus Dresden, Center of Clinical Neuroscience, Dresden, Germany; 2 Albert-Ludwigs-Universitaet Freiburg, Neurologische Klinik und Poliklinik, Freiburg, Germany; 3 Georg August University, University Medical Center Göttingen, Department of Neuropathology, Göttingen, Germany; 4 PASSAUER WOLF Reha-Zentrum Nittenau, Rehabilitationsklinik für Neurologie-Geriatrie-Urologie, Nittenau, Germany; 5 Marianne-Strauß-Klinik, Behandlungszentrum Kempfenhausen, Berg, Germany; 6 University of Basel, Department of Cognitive Psychology and Methodology, Basel, Switzerland; 7 Neuropoint Patient Academy, Neurological Practice Center, Ulm, Germany; 8 DRK Kamillus-Klinik, Asbach, Germany; 9 Novartis Pharma GmbH, Nuremberg, Germany; 10 University of Muenster, Department of Neurology, Muenster, Germany; Universite Lyon 1, FRANCE

## Abstract

**Background:**

So far, clinical studies in primary progressive MS (PPMS) have failed to meet their primary efficacy endpoints. To some extent this might be attributable to the choice of assessments or to the selection of the study population.

**Objective:**

The aim of this study was to identify outcome influencing factors by analyzing the design and methods of previous randomized studies in PPMS patients without restriction to intervention or comparator.

**Methods:**

A systematic literature search was conducted in MEDLINE, EMBASE, BIOSIS and the COCHRANE Central Register of Controlled Trials (inception to February 2015). Keywords included PPMS, primary progressive multiple sclerosis and chronic progressive multiple sclerosis. Randomized, controlled trials of at least one year’s duration were selected if they included only patients with PPMS or if they reported sufficient PPMS subgroup data. No restrictions with respect to intervention or comparator were applied. Study quality was assessed by a biometrics expert. Relevant baseline characteristics and outcomes were extracted and compared.

**Results:**

Of 52 PPMS studies identified, four were selected. Inclusion criteria were notably different among studies with respect to both the definition of PPMS and the requirements for the presence of disability progression at enrolment. Differences between the study populations included the baseline lesion load, pretreatment status and disease duration. The rate of disease progression may also be an important factor, as all but one of the studies included a large proportion of patients with a low progression rate. In addition, the endpoints specified could not detect progression adequately.

**Conclusion:**

Optimal PPMS study methods involve appropriate patient selection, especially regarding the PPMS phenotype and progression rate. Functional composite endpoints might be more sensitive than single endpoints in capturing progression.

## Introduction

Among patients with multiple sclerosis (MS) about 10% present with primary progressive MS (PPMS) [1]. The PPMS patients exhibit chronic progression from diagnosis and do not experience distinct relapses, distinguishing them from patients who develop secondary progressive MS (SPMS) after an initial relapsing-remitting phase of disease [1–3]. Compared with relapsing-remitting MS (RRMS), patients with PPMS usually have fewer brain T2 lesions and gadolinium (Gd)-enhanced T1 lesions, but more spinal cord atrophy and T2 lesions in the spinal cord [4, 5]. However, clinical findings and MRI results are not entirely consistent. Isolated relapses in some studies of PPMS suggest inflammatory activity and even Gd-enhanced lesions have been observed [6, 7]. This indicates that the underlying pathology of progressive disease courses is not well understood. It is too simplistic to dichotomize the causes of progressive and relapsing disease into “neurodegeneration” and inflammation [4]. Notably, it has been proposed recently that progressive MS phenotypes should be classified according to both activity and progression status, i.e. “active and with progression“, “active and without progression” or “not active but with progression” and “not active and without progression” [3].

No drugs are approved for the treatment of PPMS. Treatments for RRMS, e.g. glatiramer acetate (GA) or interferon-beta (IFN-beta) preparations, seem ineffective in PPMS. Up to now, no clinical study in PPMS has met its primary efficacy endpoint (e.g. time to confirmed disease progression). Even with respect to secondary outcomes, drugs under assessment rarely suggest a benefit over placebo [4]. Reasons for this may be that the outcome measures used were unable to capture appropriately the clinical progress of the disease or that the study duration was too short to measure progression, which generally manifests over a long period of time. Of course, failure may also be attributable to a lack of efficacy. The physiopathology of PPMS is poorly understood, so disease-modifying therapies that are effective in RRMS may be ineffective in PPMS because of underlying mechanistic differences between the disease phenotypes.

Moreover, even if a drug was effective in PPMS, shortcomings in the study methods could prevent a trial from demonstrating a therapeutic effect. That could relate to the chosen endpoints and outcome measures, the definition of the patient population or the study duration. The study elements chosen up to now might thus have been unsuitable to prove any therapeutic effect in PPMS patients.

In summary, study methods are critical to the evaluation of a drug’s efficacy. For example, if an outcome measure fails to detect the deterioration in the placebo group, as was seen in the PROMiSe trial, either the outcome measure or the population is inappropriate to evaluate the superiority of an active compound [6]. To develop methods suitable for future PPMS trials, it is important to scrutinize patient characteristics and disease phenotypes among studies conducted so far. Shortcomings in these studies such as endpoints specified or the trial duration need to be identified and learned from. This systematic review therefore aims to identify possible outcome influencing factors by analyzing in detail the design and methods of previous randomized studies in PPMS patients and by contrasting baseline characteristics and outcomes without restriction to intervention or comparator. By this it is intended to generate insights for future study planning especially with respect to selection of study patients, suitable assessments of disability progression and alterations within the central nervous system.

## Methods

Published studies of treatment efficacy in PPMS were evaluated to gather information about both the disease course and the sensitivity of the outcome measures used to monitor disease progression. Common parameters were evaluated with respect to their suitability for PPMS studies. A review protocol had not been developed in advance for this systematic review.

### Literature Search

To identify relevant studies, a systematic literature search was conducted by an information specialist. Databases included MEDLINE, EMBASE, BIOSIS and the COCHRANE Central Register of Controlled Trials. All databases were searched without any general restrictions with respect to language, publication type (i.e. conference proceedings were included) or date (i.e. all databases where searched from inception to present). The last search was run on 05 February 2015. Filters for randomized controlled trials were applied as part of the search strategy. Keywords used were: PPMS, primary progressive multiple sclerosis, chronic progressive multiple sclerosis and their respective truncations. The following search strategy was used, presented for the search in MEDLINE: 1) Multiple Sclerosis, Chronic Progressive”[Mesh], 2) “Multiple Sclerosis”[Mesh:NoExp], 3) Multiple-sclerosis, 4) #2 OR #3, 5) (PPMS OR PP-MS OR MS-PP OR PP-multiple-sclerosis), 6) (progressive OR progredien*) AND (primary OR chronic), 7) #4 AND (#5 OR #6), 8) #1 OR #7, 9) #8 Filters: Clinical Trial; Clinical Trial, Phase I; Clinical Trial, Phase II; Clinical Trial, Phase III; Clinical Trial, Phase IV; Comparative Study; Controlled Clinical Trial; Multicenter Study; Observational Study; Randomized Controlled Trial, 10) #8 AND (trial OR study), 11) #10 AND (therap* OR treat*), 12) #10 AND (random* OR placebo OR controlled OR double-blind OR doubleblind) 13) #9 OR #11 OR #12. This search provided 1,303 results. The adapted strategy for the other databases and the number of results per search can be found in detail in S1 Table.

### Study Selection and Appraisal

Abstracts were pre-screened by an information specialist. Selected abstracts were again screened by a further reviewer to identify possibly relevant full text publications. Study eligibility was finally assessed based on the full text. In case no full text was available, abstracts were used for the decision process. The following criteria were applied: 1) Only randomized controlled trials were eligible, 2) the study either had to be restricted to PPMS patients or in case of inclusion of a mixed MS population, PPMS-subgroup baseline data and some outcome data had to be available, and 3) study duration had to be at least one year to avoid exclusion of too many relevant studies, while allowing an adequate follow-up period over which disability progression might be detectable. No restrictions with respect to interventions, comparators or outcomes were defined. Further, publications in any language and of any publication status or year of publication were eligible. Studies meeting these criteria were included in the detailed data evaluation.

Studies that reported subgroup data for PPMS without providing information on subgroup baseline characteristics were excluded from further analysis but their results are described in the text for completeness, if, at least some PPMS-specific outcome data were available.

Duplicate publications of the same studies were identified as far as possible by cross-checking author names, study features, sample sizes, time of study conduction or outcomes. In case a full text publication was available, conference abstracts on the same study were disregarded. Multiple full text publications of one study were included in case they provided relevant additional information on baseline characteristics, outcomes or add to the interpretation of the primary manuscript.

The methodological quality of the studies and the associated risk of bias at study level were assessed by a biometrics expert. The following questions adapted from the Cochrane risk of bias assessment recommendations and additional aspects of interest were considered: 1) Are inclusion/ exclusion criteria clearly stated, 2) is study design appropriate to answer the study objectives, 3) is randomization discussed, 4) is allocation concealment discussed, 5) were study participants and personnel blinded, 6) were outcome assessments blinded 7) is sample size discussed, 8) are all pre-specified outcomes reported, 9) how is missing data handled, 10) was the analysis performed on the ITT population, 11) are there any further aspects that might bias results?

### Data Extraction

Data were extracted from full text publications. In case no full text was available, abstracts were used. No unpublished data was available at the time of this analysis. Data were initially extracted by one person. The collection was 100% quality-checked by a second person extracting the data independently into a shell table designed from the initial extraction. Disagreements were identified by a third person and resolved by consolidation by the two reviewers. The corresponding authors of the PROMiSe and the OLYMPUS study were emailed for more information. However, to date no further data was received. Raw data of the study reported by Poehlau et al. were available to the authors of the present systematic review, but did not add to the information published. The authors expected the informative value of additional data of the study reported by Leary et al. to be limited due to the small sample size of the study. Consequently, no additional data was requested from Leary et al.

### Outcomes of Interest

Basic study elements and baseline EDSS of all PPMS studies not included in the further analysis were reported for completeness. Of those studies identified for further evaluation, study features (study duration, PPMS definition, patient selection with respect to MS phenotype, age and EDSS) and population demographics (gender, age, MS phenotype, EDSS, Gd-lesion status) were evaluated to identify characteristics that might have influenced efficacy outcomes. Outcomes considered were disability progression measured using the Expanded Disability Status Scale (EDSS) or the MS Functional Composite (MSFC; including the subdomains), T2 lesion volume, T1 lesion volume, number of Gd-enhanced lesions, and brain volume. Results of the studies are presented based on the summary measures used in the respective publication (i. e. HR for the primary endpoints). No meta-analysis was performed.

### Publication Bias

Publication bias was not evaluated as no meta-analysis was performed. Further, all studies had a negative primary outcome so that it was assumed that publication bias with respect to selective reporting of positive results was no issue.

## Results

### Study Selection

**Fig 1:**
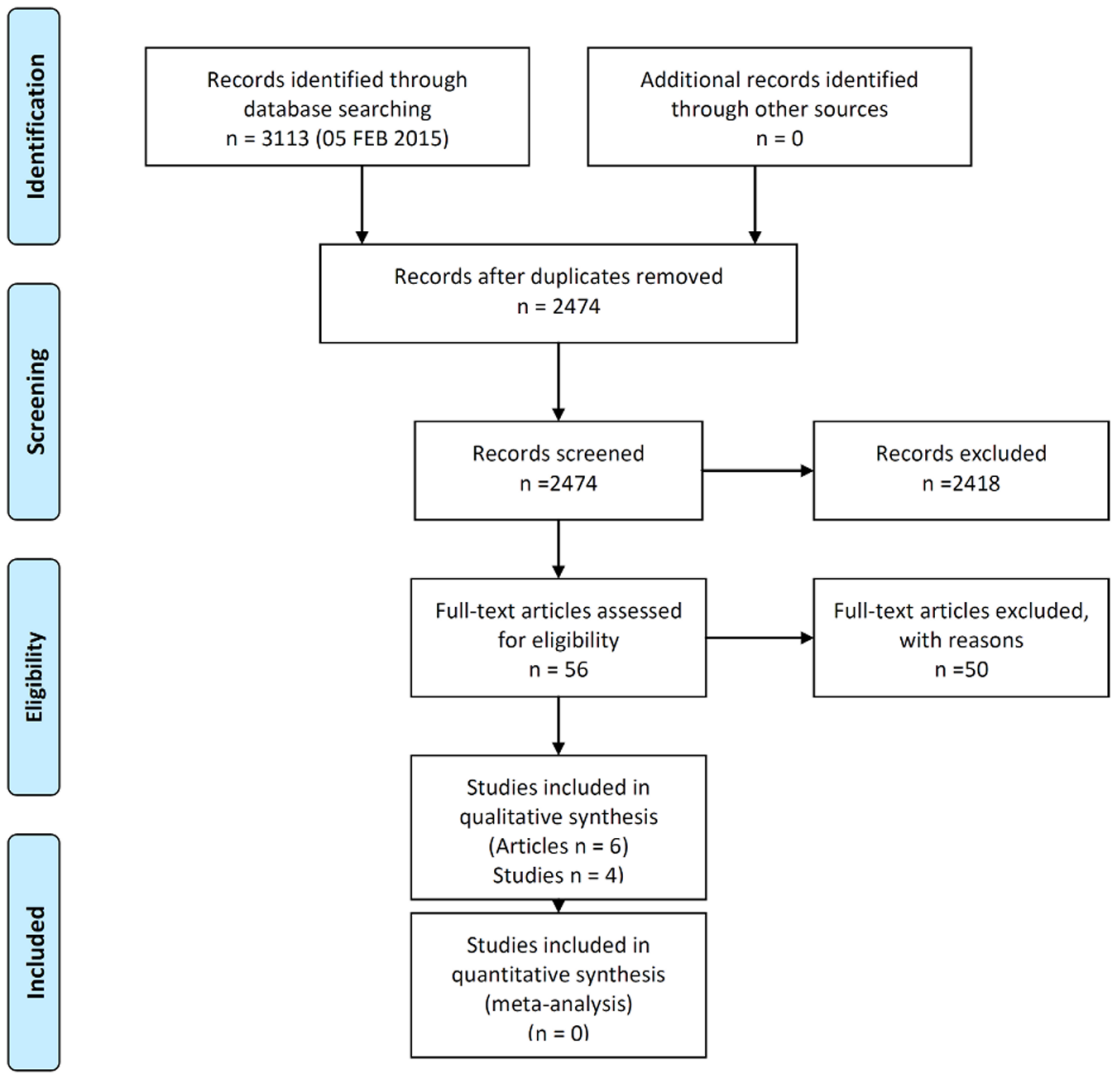
PRISMA Flow-chart (From: Moher D, Liberati A, Tetzlaff J, Altman DG, The PRISMA Group (2009). Preferred Reporting Items for Systematic Reviews and Meta-Analyses: The PRISMA Statement. PLoS Med 6(6): e1000097).

Of 52 studies identified, only four fulfilled the criteria for further evaluation: 18 studies were excluded because they were uncontrolled and 27 were excluded because PPMS-specific data were missing. Those studies missing data and most of the uncontrolled, single-arm studies either were conducted in a mixed PPMS/SPMS population, or included all types of MS without group-wise analyses. Some of the early studies in these groups referred to their study populations as “chronic progressive”. Of the remaining three studies, two were excluded because only published abstracts were available (these provided insufficient information for a further assessment), and one study was excluded because the study duration being less than one year (Fig 1).

Study and patient characteristics of the 52 prospective, interventional studies identified are presented in Table 1 (excluded studies) and Table 2 (studies selected for analysis). All studies included reported the study duration, MS phenotype, inclusion criteria with respect to age and EDSS range, gender distribution, mean age and EDSS at baseline, disease duration and Gd-lesion status [6–9]. The published results of those studies selected for analysis are summarized in Tables 3–7. All studies reported disability progression as their primary endpoint. Three of the four studies reported MSFC or subscale results and MRI outcomes, i.e. T2 lesions, T1 lesions, enhanced T1 lesions or brain volume/atrophy [6, 7, 9].

**Table 1:** Studies excluded from further evaluation.

	Study drug	N active/ placebo or comparator	Study duration (yrs)	MS subtypes	Proportion of PPMS (%)	Inclusion criterion age (yrs)	Inclusion criterion EDSS	EDSS at baseline (range or mean±SD)	Reason for exclusion
**Controlled studies not included in the analysis**									
Nabavi 2014 [33]	ASCT (2 regimen)	30 cross-over	1	iP,R,S	n/a	18–55	3.0–6.5	n/a	A
Schreiber 2014 [34]	Erythropoetin	26/26	0.5	P,S	n/a	19–60	n/a	4.0–6.5	A,B
Zajicek 2013 [14]	Dronabinol	329/164	3	P,S	39	18–65	4.0–6.5	5.9±0.69	A
Filli 2013 [35]	Fampridin	61	n/a	P,R,S	11	n/a	n/a	Median 4.5	B
Mostert 2013 [36]	Fluoxetine	20/22	2	P,S	31	18–65	3.5–6.5	4.0–6.5	A
Vermersch 2012 [37]	Masitinib	24/6	1	P,rfS	40	18–60	2.0–6.5	4.9±1.2	A
Karpha 2010 [38]	Erythropoetin	21^a^	0.4	P	100	n/a	n/a	n/a	B
Montalban 2009 [39]	IFN-beta 1b	36/37	2	P,tMS	67	18–65	3.0–7.0	3.0–7.0	A
Hellwig 2006 [40]	Triamcinolone/ Mitoxantrone	34/30	1	P,S	22	n/a	< 7.5	n/a	A
Warren 2006 [41]	MBP8298	16/16	2	P,S	31	n/a	3.0–7.5	3.5–7.5	A
Rossini 2001 [42]	4-Aminopyridine	54 cross-over	1	P,S	12	n/a	n/a	4.0–7.5	A
Rice 2000 [43] Filippi 2000 [44, 45]	Cladribine 2 groups	53/52/54	1	P,S	30	21–60	3.0–6.5	5.6	A
Beutler 1996 [46]	Cladribine	51	2	CP	n/a	n/a	n/a	n/a	A
British and Dutch Azathioprin Study Group 1988 [47]	Azathioprine	174/180	3	P,R,S	14	15–50	≤ 6	≈3.7	A
Noseworthy 1998 [48]	Sulfasalazine	103/96	3	P,R,S	13	≥ 18	1.0–4.0	2.5	A
Bosco 1997 [49]	Idebenone	11/12	0.7	CP	n/a	18–60	3.0–6.0	3.0–6.0	A
Cook 1997 [50]	TLI	24/22	3	CP	n/a	n/a	3.5–6.5	≈5.7	A
Goodkin 1995 [15]	Methotrexate	31/29	2	P,S	30	21–60	3.0–6.5	2.5–6.5	A
Cazzato 1995 [51]	Methylprednisolone	35 cross-over	0.25	P	100	n/a	n/a	n/a	D
Wiles 1994 [52]	TLI	14/10	2	P,PR	25	20–60	≤6.5	6.15±1.01	A
Milligan 1994 [53]	Isoprinosine	25/27	2	P,R,S	8	n/a	< 5.5	≈2.9	A
Sipe 1994 [54]	Cladribine	24/24	1	CP	n/a	n/a	n/a	≈4.7	A
Bornstein 1991 [55]	Glatiramer acetate	51/55	2	CP	n/a	20–60	2–6,5	≈5.6	A
Canadian Cooperative MS Study Group [56]	Cyclophosphamide Cyclophosphamide + plasma exchange	55/57/56	1	P, PR	n/a	≥15	4–6.5	≈5.7	A
Kastrukoff 1990 [57]	Lymphoblastoid IFN	50/50	2	CP	n/a	n/a	≤ 7.0	6.0	A
Cyclosporine MS Study Group 1990 [58]	Cyclosporine	273/274	2	CP	n/a	18–55	3.0–7.0	5.4	A
LaMantia 1998 [59]	Cyclophosphamide (3 regimen)	17/15/21	2	P,S	19	n/a	n/a	≈6.7	A
Cook 1986 [60]	TLI	20/20	2	CP	n/a	20–60	4.0–8.0	≈6.5	A
Gordon 1985 [61]	Plasma exchange	10/10	0.5	CP	n/a	n/a	n/a	3–8	A
Khatri 1985 [62]	Plasma exchange	26/29	1	CP	n/a	n/a	n/a	≈6.5	A
**Uncontrolled studies not included in the analysis**									
Ratzer 2014 [63]	Methylprednisolone	30^b^	1	P,S	50	n/a	n/a	n/a	A, C
Muller 2014 [64]	Triamcinolone	21^b^	<0.1	P,S	29	n/a	n/a	n/a	A, C
Romme 2014 [65]	Natalizumab	24	1	P,S	50	18–55	≤ 6.5	3.5–6.5	A, C
Arun 2013 [66]	Amiloride	14^b^	3	P	100	n/a	n/a	1.5–7.0	C
Novik 2012 [67]	ASCT (2 regimen)	95	>4	P,R,S, PR	16	n/a	n/a	1.5–8.5	A, C
Kartashov 2012 [68]	ASCT	154^b^	1	P,R,S, PR	15	n/a	n/a	3.72±1.8	A, C
Bowen 2012 [69]	ASCT	26^b^	>4	P,R,S	31	n/a	5.0–8.0	5.0–8.0	A, C
Bonab 2012 [70]	ASCT	25^b^	1	PR,S	0	18–50	3.5–7.0	3.5–7.0	A, C
Guarnaccia 2012 [71]	Daptomycin	30^b^	n/a	P,R,S, PR	7	n/a	n/a	5.4	A, C
Millonig 2008 [72]	IFN-beta 1b	20^b^	1.25	P	100	n/a	n/a	3.0–6.0	C
Gironi 2008 [73]	Naltrexone	40^b^	0.5	P	100	18–65	3.0–6.5	3.0–6.5	C
Zingler 2005 [74]	Mitoxantrone	73^b^	5	P,R,S	34	n/a	n/a	2.5–7.5	A, C
Killestein 2005 [75]	Riluzole	16^b^	2	P	100	n/a	n/a	3.0–7.5	C
Zephir 2005 [76]	Cyclophosphamide	28^b^	1	P,S	36	n/a	n/a	4.0–7.0	A, C
Hellwig 2004 [77]	Triamcinolone	161^b^	n/a	CP,S,R	n/a	n/a	n/a	3.5–6.5	A, C
Hoffmann 2003 [78]	Triamcinolone	36^b^	> 0.25	P,S	39	n/a	≤ 7.5	4.0–7.5	A, C
Bowen 2003 [79]	Pirfenidone	20^b^	1	P,S	35	18–65	3.0–6.5	3.0–6.5	A, C
Lugaresi 2001 [80]	Methotrexate	20^b^	>1.5	P,S	20	n/a	n/a	4.0–8.5	A, C

ASCT: autologous stem cell transplantation; IFN: Interferon; n/a: not available; SD = standard deviation; TLI: Total lymphoid irradiation; yrs: years.

^a^: group size not specified

^b^: single arm study

MS subtypes: CP = chronic progressive; P = primary progressive MS; iP = primary progressive MS with inflammatory activity; PR = progressive relapsing MS; R = relapsing remitting MS; S = secondary progressive MS; rfS = relapse-free secondary progressive MS; tMS = transitional MS;

Reasons for exclusion: A = insufficient PPMS subgroup data available; B = only Conference Abstract available, insufficient design information and group data for further evaluation; C = single-arm study; D = study duration < 1 year.

**Table 2:** Studies included in the further evaluation process.

	Study drug	N active/ placebo	Study duration (yrs)	PPMS only	Inclusion age (yrs)	Inclusion EDSS
**Studies included in the analysis**						
Wolinsky 2007 [6] PROMiSe	Glatiramer acetate 20mg, s.c.	627/316	3	yes	30–65	3.0–6.5
Hawker 2009 [7] OLYMPUS	Rituximab 1,0g, i.v.	292/147	2	yes	18–65	2.0–6.5
Poehlau 2007 [8]	IVIg 0,4g/kg body weight, i.v.	17/17^a^	2	no^b^	18–65	3.0–7.0
Leary 2003 [9]	IFN-beta 1a 30μg, i.m.	15/15/20	2	yes	18–60	2.0–7.0
	IFN-beta 1a 60μg, i.m.					

IVIG: intravenous immunoglobulin; IFN: Interferon; yrs: years.

^a^: sample size for PPMS patients only.

^b^: PPMS subgroup data available.

**Table 3:** Inclusion criteria with respect to PPMS definition.

	Disease duration	Presence of disability progression before inclusion	PPMS diagnosis
PROMiSe [6]	Not specified.	Progressive neurological symptoms including evidence of myelopathy for at least 6 months before the screening visit.	PPMS diagnosis according to the criteria defined by Thompson et al. [11], confirmed by the principal investigator at each study site.
			Evidence of pyramidal damage on neurological examination, including a Functional System score for the pyramidal system of 2 or greater.
			Evidence of multilevel (disseminated) central nervous system disease based on objective evidence from neurological examination alone or supplemented by findings on MRI or visual- or auditory-evoked potentials.
OLYMPUS [7]	≥ 1 year	Not specified.	PPMS according to 2001 McDonald criteria.
			Functional Systems scale score of ≥ 2.0 for the pyramidal system or gait impairment due to lower extremity dysfunction.
			Presence of IgG oligoclonal bands or elevated CSF IgG index, or both.
Poehlau 2007 [8]	>2 years	Deterioration of ≥ 0.5 points on EDSS during the previous 12 months.	Clinically definite MS according to the Poser criteria.
			Clinically active PPMS or SPMS for more than 2 years.
Leary 2003 [9]	≥ 2 year	Not specified.	Progressive history without relapse or remission.
			At least two typical lesions on MRI brain or spinal cord, and oligoclonal bands in the CSF not present in serum or abnormal visual evoked potentials.

IgG: immunoglobulin G; CSF: cerebrospinal fluid.

**Table 4:** Published results—Baseline characteristics.

		Mean Age (yrs)	Gender Male/ female (%)	EDSS (mean)	EDSS (median; range)	Disease duration since diagnosis (yrs)	Disease duration since first symptoms (yrs)	Patients with Gd-enhancing lesions (%)
PROMiSe [6, 13]	GA	50.4 ± 8.4	47 / 53	4.9 ± 1.2	n/a	5.0 ± 4.9	11.0 ± 7.3	13.9
	Placebo	50.2 ± 8.1	52 / 48	4.9 ± 1.2	n/a	5.1 ± 5.4	10.7 ±7.7	14.2
OLYMPUS [7]	Rituximab	50.1 ± 9.0	52 / 48	4.8 ± 1.4	5.0 (2.0–6.5)	4.1 ± 4.2	9.2 ± 6.4	24.1
	Placebo	49.6 ± 8.7	45 / 55	4.7 ± 1.4	4.5 (2.0–6.5)	3.8 ± 4.2	9.0 ± 6.8	25.2
Poehlau 2007 [8]	IVIg	47.8 ± 8.7	59 / 41	5.4 ± 1.2	6.0 (3.5–7.0)	n/a	7.2 ± 4.3	n/a
	Placebo	48.1 ± 10.5	65 / 35	5.8 ± 1.0	6.0 (3.0–7.0)	n/a	9.7 ± 8.9	n/a
Leary 2003 [9]	IFN 30μg	46.5	67 / 33	n/a	5.5 (3.5–7.0)	n/a	8^a^	n/a
	IFN 60μg	47	47 / 53	n/a	5.5 (2.0–6.5)	n/a	8^a^	n/a
	Placebo	43	75 / 25	n/a	4.5 (2.0–7.0)	n/a	8^a^	n/a

GA: Glatiramer acetate; IVIg: intravenous immunoglobulin; IFN: Interferon; n/a: not available; yrs: years

^a^: the publication does not name, whether duration since diagnosis or since first symptom is reported. It is assumed, that the value refers to the duration since first symptom.

**Table 5:** Disability Progression.

Study	Endpoint	Timepoint	Operationalization	Active	Placebo	Hazard ratio	95%-CI	p-value
PROMiSe	Time to 3-month confirmed disability progression	Month 36	Patients with CDP	39.6%	45.2%	0.87	0.71–1.07	0.1753
OLYMPUS	Time to 3-month confirmed disability progression	Week 48	Patients with CDP	20.2%	19.3%			0,1442
	Time to 3-month confirmed disability progression	Week 96	Patients with CDP	30.2%	38.5%	0.77	0.55–1.09	0,1442
Poehlau	Time to 4-month confirmed disability progression	Week 112	Time in weeks	96.6±13.4	68.9±10.5	n/a	n/a	0.1602
	Proportion of patients with progression	Week 112	Patients with CDP	29%	71%	n/a	n/a	0.0164
Leary	Time to 3-month confirmed disability progression	Month 24	Patients with CDP	48%^a^		n/a	n/a	ns

CDP: Clinical disability progression; CI: Confidence-interval; n/a: not available.

^a^: no group-specific data available

Definition of clinical disability progression: PROMiSe: sustained EDSS increase of ≥1.0 point in patients with a EDSS score at baseline of 3.0 to 5.0, or a sustained EDSS increase of ≥0.5 in patients with a baseline EDSS score of 5.5 to 6.5. OLYMPUS: sustained EDSS increase of ≥1.0 point in patients with a EDSS score at baseline of 2.0 to 5.5 points (inclusive), or a sustained EDSS increase of ≥0.5 point in patients with a baseline EDSS score of >5.5 points. Poehlau: sustained EDSS increase of ≥1.0 point in patients with a EDSS score at baseline of ≤5.0, or a sustained EDSS increase by ≥0.5 points, in patients with an EDSS score at baseline of >5.0. Leary: sustained EDSS increase of ≥1.0 point for patients with a baseline EDSS score ≤5.0, or a sustained EDSS increase of ≥0.5 point for patients with a baseline EDSS of ≥5.5.

**Table 6:** MSFC.

Study	Scale	Operationalization	Time	Active 1	Active 2	Placebo	p-Value
PROMiSe	MSFC	n/a	n/a	n/a	n/a	n/a	n/a
OLYMPUS	MSFC	median change from baseline	week 48	-0.00	n/a	-0.05	0.057
OLYMPUS	MSFC	median change from baseline	week 96	-0-05	n/a	-0.04	0.846
OLYMPUS	MSFC	median change from baseline	week 122	-0.06	n/a	-0.10	0.089
OLYMPUS	T25FW	median change from baseline	week 48	-0.02	n/a	-0.05	0.037
OLYMPUS	T25FW	median change from baseline	week 96	-0.07	n/a	-0.11	0.076
OLYMPUS	T25FW	median change from baseline	week 122	-0.08	n/a	-0.14	0.015
OLYMPUS	9-HPT	n/a	n/a	n/a	n/a	n/a	n/a
OLYMPUS	PASAT-3	n/a	n/a	n/a	n/a	n/a	n/a
Poehlau	MSFC	n/a	n/a	n/a	n/a	n/a	n/a
Leary	Timed 10 meter walk	Median time in seconds	baseline	11	12	9.5	n/a
Leary	Timed 10 meter walk	Median time in seconds	month 12	12	13	11	n/a
Leary	Timed 10 meter walk	Median time in seconds	month 24	19	13	14	ns
Leary	9-HPT (left/right)	Median time in seconds	baseline	26.8 / 28.8	28.4 / 28.9	29.7 / 30.2	n/a
Leary	9-HPT (left/right)	Median time in seconds	month 12	27.1 / 23.6	27.9 / 28.6	29.9 / 30.3	n/a
Leary	9-HPT (left/right)	Median time in seconds	month 24	27.2 / 23.8	30.9 / 29.0	31.2 / 31.1	ns
Leary	PASAT-3	n/a	n/a	n/a	n/a	n/a	n/a

MSFC: Multiple sclerosis functional composite; 9-HPT: 9-hole peg test; T25W: Timed 25 foot walk; PASAT-3: Paced Auditory Serial Addition Test; n/a: not available; ns: not significant.

**Table 7:** MRI results.

Study	Parameter	Operationalization	Time	Active	Active	Placebo	Reduction vs. Placebo	p-Value
PROMiSe	T2 lesions	baseline adjusted volume	year 1	n/a	n/a	n/a	39%	0.1716
PROMiSe	T2 lesions	baseline adjusted volume	year 2	n/a	n/a	n/a	71%	0.0026
PROMiSe	T2 lesions	baseline adjusted volume	year 3	n/a	n/a	n/a	58%	0.1344
OLYMPUS	T2 lesions	Mean volume change (mm^3^)	week 96	1,507 ± 3,739	n/a	2,205 ± 4,306	n/a	<0.001
Leary	T2 lesions	Median absolute volume (cm^3^)	baseline	11.5	15.8	9.5	n/a	n/a
Leary	T2 lesions	Median absolute volume (cm^3^)	month 6	10.5	14.6	13.7	n/a	n/a
Leary	T2 lesions	Median absolute volume (cm^3^)	month 12	11.0	13.1	9.6	n/a	n/a
Leary	T2 lesions	Median absolute volume (cm^3^)	month 18	11.5	15.8	12.5	n/a	n/a
Leary	T2 lesions	Median absolute volume (cm^3^)	month 24	11.0	16.3	12.7	n/a	ns/ns
PROMiSe	enhanced T1 lesions	baseline adjusted number	year 1	n/a	n/a	n/a	69%	0.0022
PROMiSe	enhanced T1 lesions	baseline adjusted number	year 2	n/a	n/a	n/a	47%	0.0702
PROMiSe	enhanced T1 lesions	baseline adjusted number	year 3	n/a	n/a	n/a	6%	0.8387
Leary	T1 lesions	Median absolute volume (cm^3^)	baseline	1.3	3.3	1.2	n/a	n/a
Leary	T1 lesions	Median absolute volume (cm^3^)	month 6	1.2	3.3	1.0	n/a	n/a
Leary	T1 lesions	Median absolute volume (cm^3^)	month 12	1.4	3.3	1.3	n/a	n/a
Leary	T1 lesions	Median absolute volume (cm^3^)	month 18	2.0	3.1	1.0	n/a	n/a
Leary	T1 lesions	Median absolute volume (cm^3^)	month 24	1.7	3.6	1.6	n/a	ns/ns
OLYMPUS	brain parenchymal fraction	Mean change (cm^3^)	week 96	-10.8	n/a	-9.9	n/a	0.62
Leary	brain atrophy BBSI	median index (cm^3^)	month 0–12	8.8	9.1	12.1	n/a	n/a
Leary	brain atrophy BBSI	median index (cm^3^)	month 0–24	12.8	14.3	15.5	n/a	ns
Leary	spinal cord area	median absolute volume (mm^2^)	baseline	70.4	65.3	69.2	n/a	n/a
Leary	spinal cord area	median absolute volume (mm^2^)	month 12	69.9	64.3	69.5	n/a	n/a
Leary	spinal cord area	median absolute volume (mm^2^)	month 24	66.7	66.8	67.9	n/a	ns
Leary	ventricular volume	median absolute volume (cm^3^)	baseline	23.5	17.7	23.3	n/a	n/a
Leary	ventricular volume	median absolute volume (cm^3^)	month 12	22.9	19.9	23.8	n/a	n/a
Leary	ventricular volume	median absolute volume (cm^3^)	month 24	22.0	21.4	26.1	n/a	n/a

n/a: not available; ns: not significant.

### Study Appraisal and Risk of Bias

All studies were randomized controlled studies and the patient in- and exclusion criteria were stated. PROMiSe and OLYMPUS were multinational multicenter studies, with PROMiSe including sites from the US, Canada and Europe, and OLYMPUS including sites from the US and Canada. The study reported by Poehlau et al. was a national trial with 15 participating sites in Germany. The study reported by Leary et al. was conducted at a single center in the United Kingdom. Information on the randomization procedure was missing for PROMiSe and OLYMPUS and none of the studies discussed allocation concealment. It was unclear from the publication by Poehlau et al. whether outcome assessments were performed by blinded study personnel.

Sample size and power considerations were adequate for PROMiSe and OLYMPUS: In the PROMiSE trial, sample size was based on the assumption that 50% of the placebo-treated patients with a baseline EDSS of 3.0 to 5.0 and 20% of those with a baseline EDSS of 5.5 to 6.5 would progress within one year. Accordingly, the assumed yearly hazard ratio for survival was 0.307. Glatiramer acetate was considered to delay progression by 40% and the drop-out rate was projected to be 40%. The target sample size was 900 patients, resulting in a power of 84.5%. In the OLYMPUS study, sample size calculations were based on the assumption that progression rate would be 32% at 96 weeks. The study had a power of about 90% to detect a 50% reduction in progression. No information on the final estimation of the sample size was presented. Sample size calculation in the study reported by Poehlau et al. was based on a mixed population for the functional improvement. It was assumed that the sample size calculated for the functional improvement would be sufficient for the analysis of progression. An estimation of patient numbers by type of MS was not performed. The analysis of the PPMS subpopulation was thus not adequately powered. Leary et al. did not report any considerations on sample size. The study was exploratory and included only 50 patients in total. The study is considered not to be adequately powered.

All studies followed the ITT principle. Selective reporting of outcomes was considered to be no issue as the primary endpoints were not met. The risk of bias with respect to handling of missing data is unclear, as relevant information is missing. Of note, the PROMiSe trial was terminated early. As this was done due to lack of efficacy, the risk of bias can still be considered low. Randomization in the study reported by Poehlau et al. was not stratified by disease type, which increases risk of bias. As the study reported by Leary et al. was conducted in a single center, probably adding bias to the outcomes. No study was excluded for risk of bias, as the identification for aspects biasing results towards a negative treatment outcome are the major interest of this review. A table presentation of the risk of bias assessment at the study level can be found in the supplement. Relevant considerations at the outcome-level are therefore presented together with the respective results in the following.

### Study and Patient Characteristics

The studies analysed were all of 1 to 3 years’ duration, were randomized, placebo-controlled, and were conducted double-blind. Respectively, the PROMiSe trial by Wolinsky et al. [6] and the OLYMPUS study by Hawker et al. [7] included 943 and 439 patients, who were randomized to active treatment or to placebo in a 2:1 ratio. The studies reported by Poehlau et al. [8] and Leary et al. [9] were significantly smaller with 15 to 20 PPMS patients per treatment arm. In all studies patients were on average 45 to 50 years of age. The gender distribution in PROMiSe and OLYMPUS was well balanced, each having 50% men and women and thus being representative of the general PPMS population [10]. There were more men than women in the two smaller studies.

Inclusion criteria varied between studies with respect to the definition of PPMS. PROMiSe followed diagnostic criteria defined by Thompson et al. [11], which also served as the basis for the McDonald criteria for PPMS. These defined three levels of certainty for PPMS diagnosis: definite, probable and possible. Definite PPMS applied if all of the following criteria were fulfilled: clinical progression for at least one year, oligoclonal bands in the cerebrospinal fluid as well as positive MRI evidence or equivocal MRI evidence together with delayed visual-evoked potentials. Probable PPMS lacks either unequivocal MRI findings or oligoclonal bands in the cerebrospinal fluid and possible PPMS lacks both. The PROMiSe investigators could only verify a diagnosis of definite PPMS in about 65% of the patients. That means, about one third of the patients did not suffer from definite PPMS [12]. In OLYMPUS, the 2001 McDonald diagnostic criteria were followed and at least one year of disease history was required. Poehlau et al. did not specify the diagnostic criteria used in their study, but stated that patients had to have clinically active PPMS or SPMS for at least two years. Leary et al. stipulated a progressive history without relapse or remission in at least the preceding two years (Table 3).

### Patients’ MS Disease History

In these four trials, median baseline EDSS was in the range 4.5 to 6.0. Although the EDSS ranges were similar across studies, variation in the mean disease duration since first symptoms indicated that the study populations differed with respect to their progression rate. First symptoms occurred 11 years before study entry in PROMiSe and about 9 years before enrolment in OLYMPUS. Time from first symptoms to baseline was about 8 years in the PPMS cohorts reported by Poehlau et al. and by Leary et al. The proportion of patients with Gd-enhanced lesions at baseline differed remarkably between the two large studies: 14% of patients presented with Gd-enhanced lesions in PROMiSe, while in OLYMPUS the proportion was 25% (Table 4). Pretreatment status was only reported for the OLYMPUS: 30% of patients had received prior treatment and stopped more than 90 days before enrolment; 5% of patients stopped treatment during the 90 days before study entry. The type of medication used was not reported.

### Outcome Measures

#### Disability

**Fig 2:**
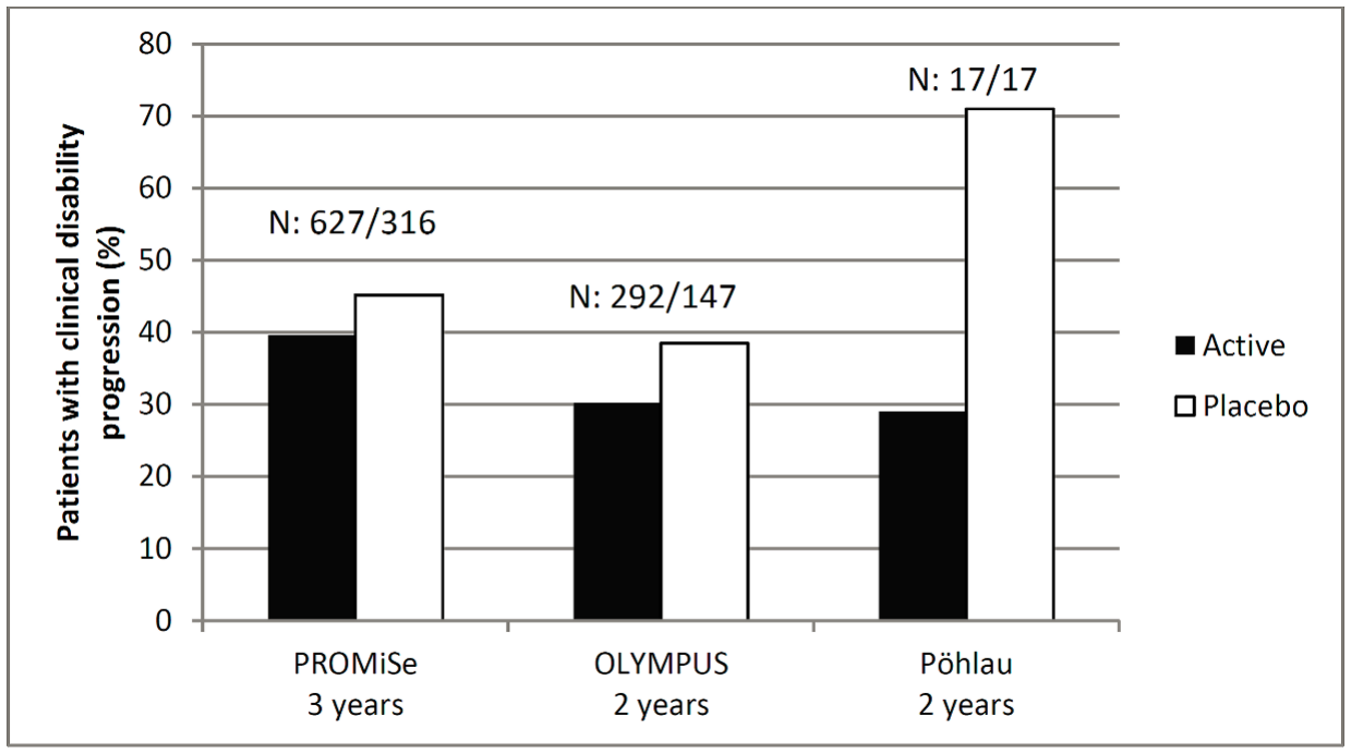
Proportion of patients with clinical disability progression; N = number of patients in the respective group; Definition of clinical disability progression: sustained EDSS increase of ≥1.0 point in patients with an EDSS score at baseline of 3.0 to 5.0, or a sustained EDSS increase of ≥0.5 in patients with a baseline EDSS score of 5.5 to 6.5 (PROMiSe); sustained EDSS increase of ≥1.0 point in patients with an EDSS score at baseline of 2.0 to 5.5 points (inclusive), or an EDSS increase of ≥0.5 point in patients with a baseline EDSS score of >5.5 points (OLYMPUS); sustained EDSS increase of ≥1.0 point in patients with an EDSS score at baseline of ≤5.0, or a sustained EDSS increase by ≥0.5 points, in patients with an EDSS score of >5.0 at baseline (Poehlau et al.).

In all studies the primary endpoint was defined as the time to sustained clinical disability progression (CDP), but no significant treatment effect was reported for this endpoint in any of the four studies (Table 5 and Fig 2).

**Fig 3:**
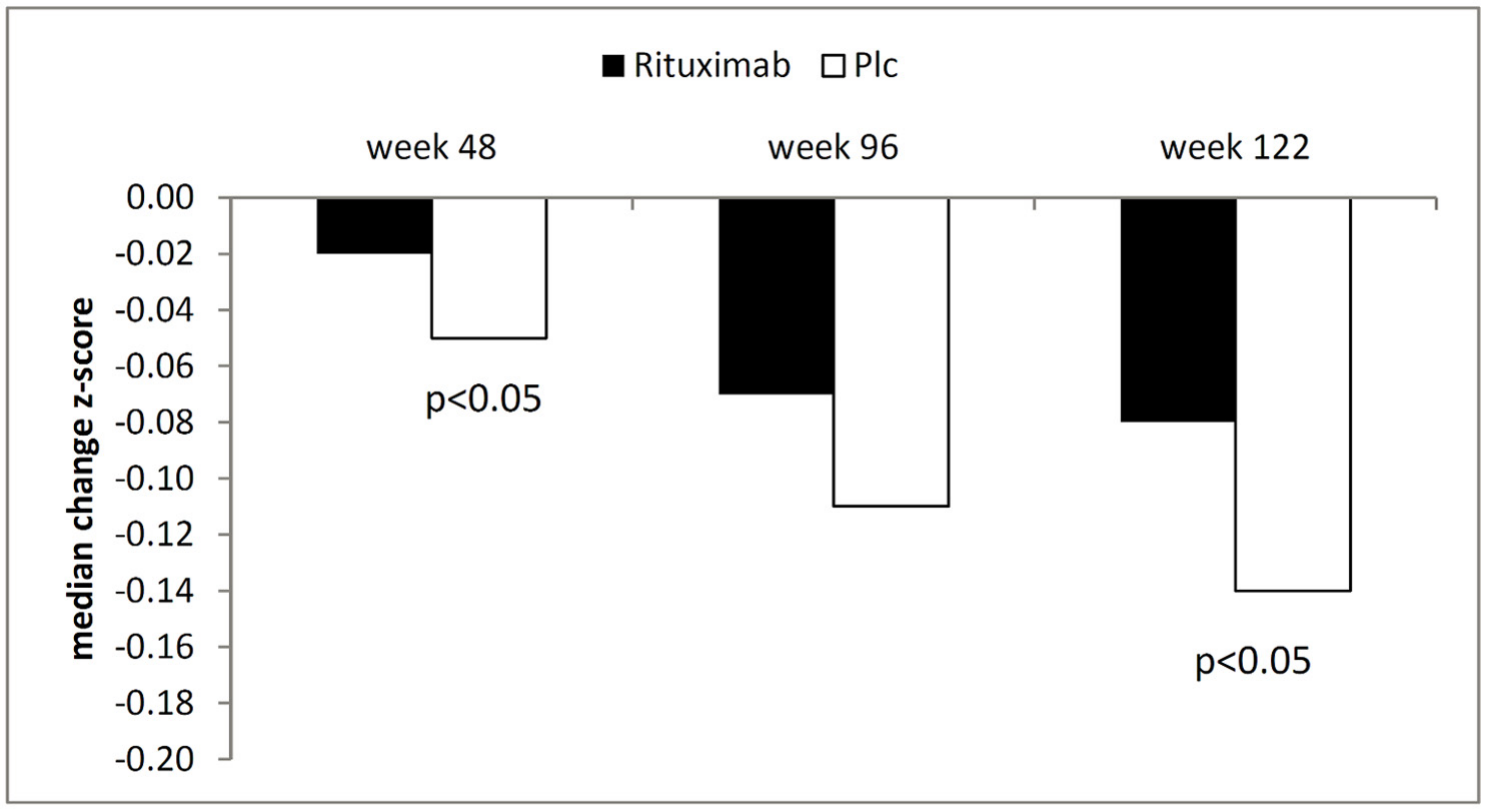
Median change from baseline in T25W in the OLYMPUS study (reported by Hawker et al. as z-score; the Z-score is calculated by subtracting the baseline mean from each individual test result and then dividing by the standard deviation of the baseline values to obtain a standardized score for each individual); * p<0.05 compared to placebo.

Changes from baseline in EDSS and MSFC scores were not statistically significant between treatment groups in PROMiSe [6]. In OLYMPUS, deterioration in the timed 25-foot walk (T25W) was slower with rituximab than with placebo, and this effect reached statistical significance at weeks 48 and week 122. No statistically significant effect was observed in OLYMPUS with respect to changes in MSFC scores (Table 6 and Fig 3).

In a small population, Poehlau et al reported that proportionately fewer patients receiving intravenous immunoglobulin (IVIg) than placebo had disability progression based on change in EDSS score [8]. However, the proportion of patients experiencing disability progression in the placebo group was considerably greater than that observed in the placebo groups of the other studies examined (Table 5 and Fig 2). Moreover, this treatment effect was not consistent with the analysis of time to disability progression also reported by Poehlau et al., which showed no between-group differences. Patients who discontinued this study were counted as having disease progression, which may have biased the results in favour of a significant treatment effect. Further, patients in this study had a similar baseline EDSS but a shorter disease duration than patients in the other studies analysed, indicating that in relative terms, patients in the study by Poehlau et al. had a higher progression rate. MSFC was not assessed. [8].

**Fig 4:**
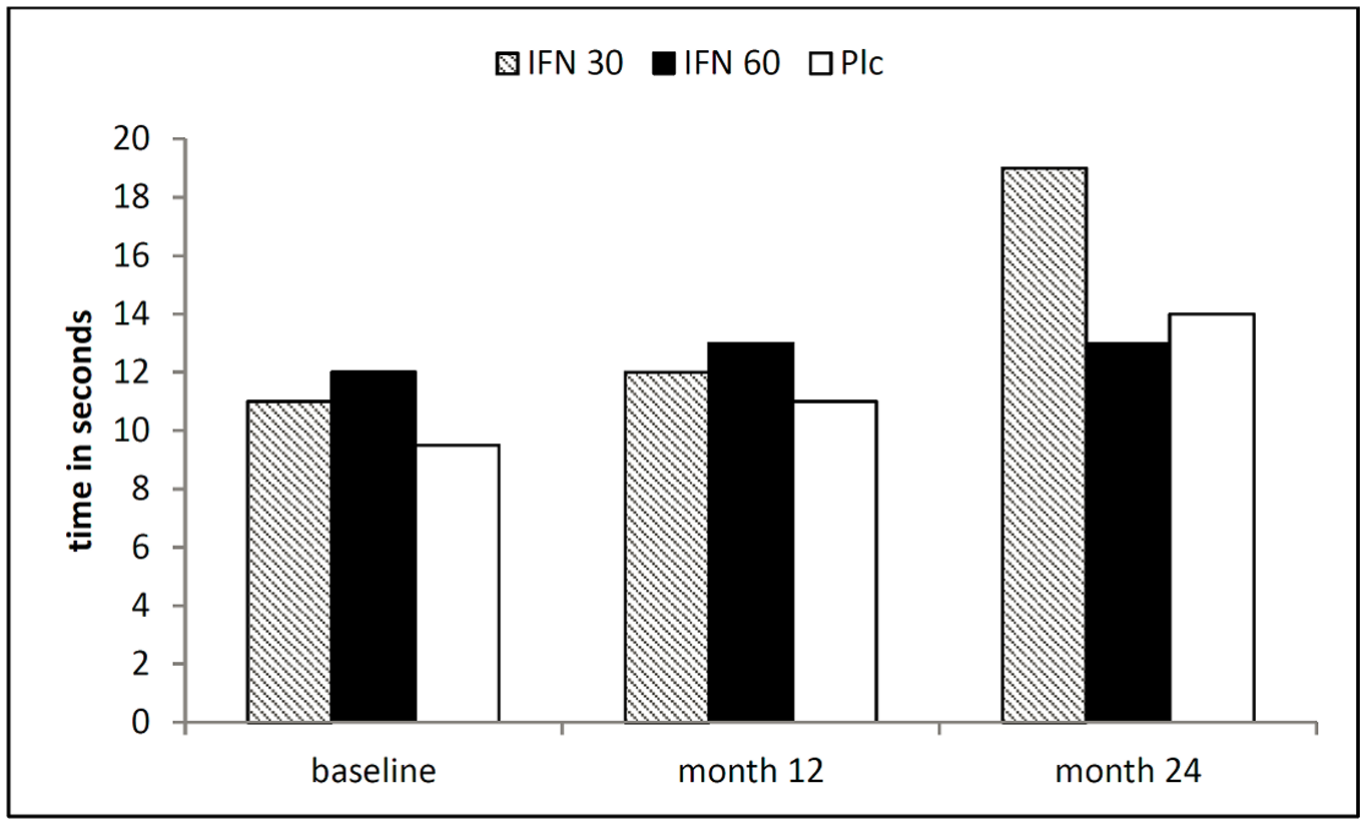
Results from the timed 10-meter walk in the study by Leary et al. (median time in seconds).

In the study reported by Leary and colleagues, which examined the effects of intramuscular IFN-beta 1a at two dosages in a small patient population, two subscores of the MSFC were assessed: the timed 10-meter walk (comparable to the T25W) and the nine-hole peg test (9-HPT; Table 6 and Fig 4). The placebo and low-dose IFN-groups showed increases in the timed 10-meter walk, whereas results were stable in the high-dose IFN-group. Results for EDSS were not reported, except within the analysis of time to disability progression [9].

In PROMiSe, significant subgroup-specific effects were observed. Men had a 30% lower risk of disability progression on glatiramer acetate than on placebo, with the effect being statistically significant [6]. However, this treatment effect was sensitive to variations in the statistical model used. When time on study was included as a covariate the effect was no longer significant [13]. The investigators reviewed the literature for evidence of gender-based differences with respect to glatiramer acetate treatment in both PPMS and RRMS. Neither the analysis nor the literature suggested gender effects on MRI-outcomes in PPMS. The literature did also not support gender-specific differences on relapse rate or MRI-outcomes in RRMS [13]. According to Wolinksy et al., the probability of progression in the placebo arm was 30% lower in women than in men, indicating that men progressed faster than women.

Subgroup effects were also found in OLYMPUS. Among patients with Gd-enhanced lesions at baseline, the rituximab-treated group showed a delay in time to confirmed disability progression compared with the placebo group. Furthermore, patients with baseline Gd-enhanced lesions, who were also less than 51 years of age, had a significantly reduced risk of disability progression on treatment (rituximab vs. placebo, HR = 0.33; p = 0.0088). In contrast, no significant treatment effect was observed among those aged 51 years or older [7]. Regardless of age, the median time to progression among patients with Gd-enhanced lesions treated with placebo was in the range 70 to 85 weeks, but could not be estimated among patients in this subgroup treated with rituximab. In fact, median time to progression could not be estimated for any patients lacking Gd-enhanced lesions at baseline. That means that fewer than 50% of patients met the definition of clinical disability progression during the course of the study. Therefore, for the majority, the median time to progression was greater than 96 weeks [7].

Two further studies provided information on disability progression from PPMS subgroups. These studies were not analysed in detail because baseline characteristics for the subgroup were not reported. However, the reported subgroup results are presented briefly for completeness: The CUPID study [14], which assessed the effect of dronabinol vs. placebo in patients with PPMS and SPMS, showed no difference in the risk of disability progression within the PPMS subgroup (HR = 1.08; p = 0.74). Additionally, a second study [15] that compared methotrexate with placebo, found no between-group difference in the proportions of PPMS patients with disability progression (methotrexate, 42.9%; placebo, 63.6%; p = 0.630).

### MRI

The treatment effect on T2 lesion volume in PROMiSe was inconsistent year on year (Table 7). Although between-group differences were not significant in years 1 and 3, there was a significantly smaller mean increase in T2 lesion volume in year 2 associated with glatiramer acetate than with placebo [6]. There was also a significant between-group difference in the change in T2 lesion volume from baseline to week 96 in OLYMPUS [7]. It remains unclear whether effects on T2 lesion volume are attributable to changes in just a few patients. Poehlau et al. did not report T2 lesion data [8] and no significant changes were observed in the study reported by Leary et al. [9].

No treatment effects on T1 lesions were reported in any of the studies included in this review. Brain atrophy was only reported for OLYMPUS (measured by change in brain parenchymal fraction) and by Leary et al. (measured by the brain boundary shift integral). No significant treatment effects on brain atrophy were observed with rituximab and intramuscular IFN-beta 1a, respectively. In addition, intramuscular IFN-beta 1a had no effect on changes in spinal cord area or ventricular volume [7, 9].

Considering sub-group analyses, no gender-based differences in MRI assessments were found in the PROMisE study [13]. However, in OLYMPUS, difference in treatment effects were seen when subgroups were dichotomized by age and Gd-enhanced lesion status. Two subgroups saw significant benefit from rituximab treatment with respect to relative reduction in total T2 lesion volume:

1. Younger patients (< 51 years of age) with Gd-enhanced lesions at baseline (relative risk reduction 61.6%, p = 0.021, rituximab vs. placebo) and
2. Older patients (≥ 51 years of age) without Gd-enhanced lesions at baseline (relative risk reduction 34.8%, p = 0.022, rituximab vs. placebo).

## Discussion

None of the studies evaluated provided efficacy evidence for any PPMS treatment, neither with respect to disability outcomes nor to MRI outcomes. With respect to the studies reported by Poehlau et al. and Leary et al., the small size of the studies has to be kept in mind when the findings are interpreted for their general clinical relevance. Nevertheless, these two studies provided important information with respect to treatment evaluation in PPMS patients. Furthermore, their results are in line with the two larger studies assessed in the present review. However, although the PROMiSE and the OLYMPUS trial were larger in size, the absence of significant delay in the analysis of disability progression should be interpreted with caution with respect to their relevance for clinical treatment decisions.

The fact that all drugs so far assessed in PPMS have failed to meet the primary outcome, may be because drugs known to be effective in RRMS are inherently ineffective in PPMS. RRMS is characterized by a disrupted blood-brain barrier, leukocyte invasion and focal acute inflammation [16]. The physiopathology of PPMS remains unclear. However, in contrast to RRMS, the blood-brain barrier, as evidenced by Gd-enhancement, is largely closed within PPMS. It is characterized by a chronic inflammatory disease course with consecutive damage to myelin, oligodendrocytes, axons and neurons. Diffuse inflammation with microglial activation in the normal-appearing white matter and cortical demyelination can also be observed [5, 16]. With respect to these differences in the underlying pathology, RRMS drugs might thus simply be unsuitable for treating PPMS.

However, the present evaluation points to the importance of appropriate study planning for future drug development with respect to the choice of assessments and patient selection. The majority of studies conducted so far did not selectively evaluate PPMS patients. On this basis, it is not possible to make a reliable assessment of a drug’s efficacy in PPMS. Selective inclusion of patients that accurately distinguishes between different progressive disease courses is therefore a requirement for future studies. Furthermore, treatment failure might also be attributable to sub-therapeutic concentrations of the drug reaching the target area, either through inadequate dosing or the inability of the substance to access the central nervous system. With respect to accessibility it is also important to consider the drug’s free versus its bound form and the specific target.

However, even a drug especially designed for PPMS could fail in a trial because of inappropriate selection of sample size, study duration, endpoints or population. The lack of data on PPMS treatment impedes reliable sample-size calculations. For example, natural history data are inconsistent regarding progression rates. In a Calgary cohort, a median time of 9 years was reported for progression to an EDSS score of 6.0 [17], compared with 14 years in a British Columbia cohort [18]. Furthermore, in an analysis of patients from South-East Wales, the time to progression from an EDSS score of 5.0 to 6.0 was notably higher than for progression from a score of 4.0 to 5.0 [19], which has to be considered when calculating sample size. Although this was accounted for in the PROMiSe trial, there was an unexpectedly low progression rate among patients with a baseline EDSS score in the range 3.0 to 5.0 (50% of the study population). Within the first 12 months of treatment, only 16.1% of these patients progressed and the sample-size calculation was based on an assumption that 50% would progress in that time. Among patients with a baseline EDSS score in the range 5.5 to 6.5 the observed progression rate (19.3%) matched the estimated rate (20%).

Considering the study reported by Poehlau et al., patients presumably had a particularly high progression rate at enrolment that may have biased the analysis of disability progression towards significance: patients receiving placebo also showed a higher rate of disability progression than those in other relevant trials. Of note, only in the study reported by Poehlau et al. was disease progression a requirement for study entry. It might therefore be assumed, that the other studies enrolled a substantial number of patients with relatively stable disease. The mean baseline EDSS score was approximately 5.5 and mean disease duration was 7.2 years in the IVIg group and 9.7 years in the placebo group. In contrast, the mean baseline EDSS score in OLYMPUS and PROMiSe was less than 5.0 and the disease duration in the range 9.0 to 11 years. This means that patients in the study reported by Poehlau et al. progressed to higher EDSS values in a shorter time than did patients in OLYMPUS and PROMiSe. With regard to the study reported by Poehlau et al., it must be noted that the sample size was small, thus limiting what conclusions can be drawn.

Study results are prone to misinterpretation if eligibility criteria for a PPMS study population are not clearly defined and allow for inclusion of patients with inflammatory disease activity. The obvious inclusion of patients with inflammatory disease was the primary reason why the majority of the identified studies were excluded from the present assessment. Such studies included a mixed population of PPMS and SPMS patients, and often did not distinguish between these MS types, referring only to the population as “chronic progressive”. Therefore, the importance of a clear definition of PPMS in the inclusion criteria must be stressed: Patient baseline characteristics, e.g. disability or MRI status might influence the study outcome. A significant treatment effect was seen in OLYMPUS among patients with Gd-enhanced lesions at baseline. At 25%, the proportion of patients with Gd-enhanced lesions in this study was substantially higher than in PROMiSe (14.1%) [6, 7], but in general, patients with PPMS have been reported to have lower rates of Gd-enhanced lesions than patients with other forms of MS [5]. However, variation in the incidence of Gd-enhanced lesions may arise because studies assess different phases of the disease. Hence, studies, which selectively assessed early phases of PPMS reported more Gd-enhanced lesions compared to later phases [20, 21]. Further, the MRI protocol may account for differences. More Gd-enhanced lesions are seen following a triple dose of gadolinium diethylenetriaminepentacetate (0.3 mmol/kg) than following a single dose (0.1 mmol/kg) [20, 21]. Consideration of the effect of variation in all of the disease-specific baseline characteristics must be made by an independent review committee to standardize PPMS diagnosis and thus, the eligibility of patients for enrolment.

The results of PROMiSe suggested that gender might influence study outcome and that subgroup evaluation might therefore be important. However, the PROMiSe investigators scrutinized their data in the context of the literature and found no evidence that gender influences the outcomes of PPMS patients treated with glatiramer acetate [13]. Evidence from natural history studies on PPMS regarding gender-specific disease courses is inconsistent. For example, a progression rate in men that is twice that in women has been reported [22], while analyses from the British Columbia MS database show no difference between the sexes [18]. Whether gender-specific evaluation of drug effects in PPMS should be undertaken can neither be supported nor dismissed at this time.

Currently, there are no recommendations regarding PPMS study endpoints. Efforts were made by the MSOAC, a consortium in collaboration with academic, FDA and EMA to look into the proper definition of endpoints for future trials [23]. This initiative is still ongoing. To date, the endpoints used are those typically specified in RRMS trials and it is unclear whether these are suitable for use in PPMS studies. At the moment there is little evidence for their suitability, though this may be attributable to the shortcomings already discussed. The study reported by Poehlau et al. showed a positive but inconsistent effect of treatment with IVIg, based on changes in EDSS scores [8], but this effect has not been confirmed in a larger study. IVIg is not currently indicated in PPMS in current guidelines, e.g. those of the German Neurological Society (Deutsche Gesellschaft fuer Neurologie, DGN) [24] or of the American Academy of Neurology [25]). Similarly, the effects of rituximab on T25W in OLYMPUS were inconsistent over time.

A post hoc analysis of data from the OLYMPUS trial explored the use of combinations of EDSS, T25W and 9HPT in different composite endpoints. Disability progression based on changes in EDSS score was defined as an increase of either ≥1 point or ≥0.5 points, depending on baseline score. For T25W and 9HPT, disability progression was defined as a 20% worsening from baseline [26]. This choice of rate is consistent with another report, which showed that this degree of worsening is clinically meaningful and impacts on daily life [27]. One of the composite measures, which assessed disability progression based on changes in EDSS score or T25W or 9HPT, showed a treatment effect similar to that observed when assessment was based on changes in EDSS alone, but also revealed much higher progression rates. The increased sensitivity of such a composite measure could therefore increase the statistical power of PPMS trials [26].

There is also a need for endpoints which capture patients’ perceptions of their health status. In routine practice, patients commonly report that their health deteriorates, but translating such perceptions into validated measures of health status is a problem as yet unresolved. One option may be to evaluate mobility by continuous monitoring with a smartphone app; patient-reported changes in EDSS score might also be suitable, and the MS Walking Scale could be used to quantify ambulation. Guy's Neurological Disability Scale (GNDS) was recently evaluated as a measure of disability alongside EDSS, T25W and 9-HPT: GNDS is an interview-based questionnaire that examines neurological disability from the patient’s perspective, and the study recommended using both the GNDS and T25W in assessment of progressive MS [28]. Neuropsychological outcomes like fatigue or cognitive impairment have seldom been considered as outcome measures, but both are relevant and important symptoms of PPMS so they may prove to be informative endpoints in future trials [29, 30].

As with clinical assessments, the value of MRI outcomes (particularly brain atrophy) in evaluating disease progression cannot be adequately determined based on the studies included in this review. Sample sizes were either too small or baseline information with respect to MRI activity was insufficient to draw any conclusions. In addition, it has to be acknowledged that those MRI parameters that were sensitive to treatment in OLYMPUS and PROMiSe (Gd-enhanced lesions and T2-lesions) are also those parameters that would be considered of minor relevance in PPMS in the context of the current literature [5]. Therefore, as well as questioning, whether study populations were truly representative of the PPMS phenotype, the possibility must be considered that study results may be biased significantly by a small number of PPMS patients with inflammatory disease activity. These issues remain unresolved, but should be borne in mind in future study designs. Regarding brain atrophy, evidence for its utility and validity as an outcome measure in early PPMS remains unproven, but it is an endpoint currently specified in several phase 2 studies. Hopefully, these will clarify whether this measure is of value in PPMS. Other paraclinical imaging techniques such as magnetization transfer, MR spectroscopy, high resolution MRI or optical coherence tomography may also be valuable in monitoring disease progression, but their suitability remains to be assessed in clinical studies.

The INFORMS study was designed to evaluate the efficacy and safety of fingolimod in PPMS and was completed in 2014. It attempted to address some of the issues discussed above, especially with respect to selection of the study population. To be eligible, patients had to be diagnosed with PPMS according to the 2005 revised McDonald criteria, and those with a history of relapses were excluded. Patients had to have an EDSS score in the range 3.5 to 6.0 at screening, with an increase of at least 0.5 points during the two years prior to screening. Additionally, they had to show disability progression in each of the preceding two years. Furthermore, patients’ pyramidal function score had to be at least 2.0 and their T25W had to be less than 30 seconds. This design was meant to use criteria selecting patients with active disease progression and motor impairment, while still being able to fulfill the assessments. In addition, patient eligibility regarding the diagnosis of PPMS was evaluated by an independent review committee [31]. Recently, first results of the INFORMS study were presented at international neurologists conferences. The primary endpoint analysis, i.e. time to sustained disability progression for patients treated for at least 36 months did not show a significant difference between fingolimod and placebo. Sustained disability progression was defined as either at least 20% increase in the T25W, at least 20% increase in the 9-HPT or as EDSS increase of 1 point in patients with baseline EDSS 3.5 to 5.0, and 0.5 point with baseline EDSS 5.5 or 6.0 [32]. Detailed results have not been published yet, but will further add to the evaluation of methods for PPMS studies.

In conclusion, the lack of efficacy evidence for treatments in PPMS may be because PPMS differs fundamentally from relapsing and secondary-progressive disease courses. Drugs assessed to date may not target the pathology of PPMS or may simply be unable to cross the blood-brain barrier. Development of drugs that can target these mechanisms is vital, but in addition, trials need to be designed appropriately to ensure that treatment effects do not go undetected for technical reasons. Trial methods must also ensure that the patient population is clearly defined allowing distinct MS phenotypes to be studied. Variation in progression rates of the patients must also be considered. Composite endpoints may be more sensitive to capture disease progression and treatment effects.

## Supporting Information

S1 Table PRISMA Checklist. (PDF)

S2 Table Literature Search. (PDF)

S3 Table Assessment of methodological quality and risk of bias of studies included in the detailed evaluation at Study Level. (PDF)
