# Supplementary material for: Evaluation of Study and Patient Characteristics of Clinical Studies in Primary Progressive Multiple Sclerosis: A Systematic Review
Source: PLoS One. 2015 Sep 22;10(9):e0138243. doi: 10.1371/journal.pone.0138243 (PMC4578855; doi:10.1371/journal.pone.0138243)
Supplement: S2 Table — (PDF) [file pone.0138243.s002.pdf]

S2 Table. Literature Search

Topic:
Primary Progressive Multiple Sclerosis (PPMS): Clinical Trials

Databases:
PUBM, EMBASE, BIOSIS, COCHRANE Central Register of Controlled Trials
(no general restrictions)

Search Strategy per Database:

Database PUBMED
Search platform --
Search Date 05.02.2015
Dates of Coverage
Filters ---

#	Search Terms	Results
1	"Multiple Sclerosis, Chronic Progressive"[Mesh]	1374
2	"Multiple Sclerosis"[Mesh:NoExp]	40560
3	Multiple-sclerosis	64619
4	#2 OR #3	64619
5	(PPMS OR PP-MS OR MS-PP OR PP-multiple-sclerosis)	663
6	(progressive OR progredien*) AND (primary OR chronic)	46684
7	#4 AND (#5 OR #6)	3591
8	#1 OR #7	3591
9	#8 Filters: Clinical Trial; Clinical Trial, Phase I; Clinical Trial, Phase II; Clinical Trial, Phase III; Clinical Trial, Phase IV; Comparative Study; Controlled Clinical Trial; Multicenter Study; Observational Study; Randomized Controlled Trial	813
10	#8 AND (trial OR study)	2022
11	#10 AND (therap* OR treat*)	887
12	#10 AND (random* OR placebo OR controlled OR double-blind OR doubleblind)	478
13	#9 OR #11 OR #12	1303

Database EMBASE
Search Platform Ovid
Search Date 05.02.2015
Dates of Coverage 1974 to 2015 February 03
Filters ---

#	Search Terms	Results
1	exp multiple sclerosis/	86425
2	Multiple-sclerosis.mp.	94241
3	1 or 2	94241
4	(ppms or pp-ms or ms-pp or pp-multiple-sclerosis).mp.	1275
5	((primary or chronic) and (progressive or progredien*)).mp.	65750
6	4 or 5	66169
7	3 and 6	4724
8	(randomised or randomized or double-blind or doubleblind or placebo).mp.	919872
9	((study or trial) and (therap* or treat*)).mp.	4178917
10	Clinical study/	110092
11	Exp clinical trial/	1019140
12	Exp "clinical trial (topic)"/ or exp intervention study/ or exp major clinical study/	2471359
13	Or/8-12	5945557

14	7 and 13	2392
15	Random:.tw.	967567
16	Placebo:.mp.	347981
17	Double-blind:.tw.	155296
18	Or/15-17	1187294
19	7 and 18	584
20	Conference abstract.pt.	1804149
21	14 and 20	612
22	19 or 21	1022
Database COCHRANE Central Register of Controlled Trials<br>Search Platform Ovid<br>Search Date 05.02.2015<br>Date of Coverage 1991 – January 2015		
#	Search Terms	Results
1	Multiple sclerosis.mp.	3655
2	Ppms.mp.	44
3	pp-ms.mp.	6
4	Ms-pp	2
5	pp-multiple-sclerosis	1
6	((progressive or progredien*) and (primary or chronic)).mp.	2290
7	(progressive or ppms or progredien* or pp-ms or ms-pp).ti.	1391
8	Or/2-7	3241
9	1 and 8	432
10	Exp multiple sclerosis, chronic progressive/	133
11	9 or 10	432
Database BIOSIS<br>Search platform Ovid<br>Search date 05.02.2015<br>Date of Coverage 1995 – 2015 Week 6		
#	Search Terms	Results
1	Multiple sclerosis.mp.	44101
2	Ppms.mp.	490
3	pp-ms.mp.	100
4	Ms-pp	17
5	pp-multiple-sclerosis	22
6	((progressive or progredien*) and (primary or chronic)).mp.	24087
7	(progressive or ppms or progredien* or pp-ms or ms-pp).ti.	13951
8	Or/1-6	66564
9	1 and 7 and 8	1185
10	9 and meeting.pt.	390
11	10 and (clinical or random* or therap* or treat*).af.	356
