# Supplementary material for: Evaluation of Study and Patient Characteristics of Clinical Studies in Primary Progressive Multiple Sclerosis: A Systematic Review
Source: PLoS One. 2015 Sep 22;10(9):e0138243. doi: 10.1371/journal.pone.0138243 (PMC4578855; doi:10.1371/journal.pone.0138243)
Supplement: S3 Table — (PDF) [file pone.0138243.s003.pdf]

S3 Table. Assessment of methodological quality and risk of bias of studies included in the detailed evaluation at study level

	**PROMiSe**	**OLYMPUS**	**Poehlau 2007**	**Leary 2003**
**Are inclusion/ exclusion criteria clearly stated?**	Yes,<br>low risk	Yes,<br>low risk	Yes,<br>low risk	Yes,<br>low risk
**Is study design appropriate to answer the study objectives**	RCT,<br>Low risk	RCT,<br>Low risk	RCT,<br>Low risk	RCT,<br>low risk
**Is randomization discussed?**	No discussion<br>unclear risk	No discussion<br>unclear risk	Yes, third party, random blocks<br>low risk	Yes, random blocks<br>low risk
**Is allocation concealment discussed?**	No discussion<br>unclear risk	No discussion<br>unclear risk	No discussion<br>unclear risk	No discussion<br>unclear risk
**Were study participants and personnel blinded?**	Yes<br>low risk	Yes<br>low risk	Yes<br>low risk	Yes<br>low risk
**Were outcome assessments blinded?**	Yes,<br>low risk	Yes,<br>low risk	Not discussed,<br>unclear risk	Yes (discussed for primary endpoint assessments),<br>low risk
**Is sample size discussed?**	Yes, adequate<br>low risk	At least considerations on statistical power reported,<br>low risk	Sample size calculation for mixed population (PPMS and SPMS); sample size not reached, and PPMS group very small<br>high risk	No, 50 patients enrolled, exploratory study<br>high risk
**Are all pre-specified outcomes reported?**	No protocol available, but not all mentioned endpoints reported, but as the primary outcome is negative, selective reporting is considered to be no issue<br>low risk	No protocol available, all of the described endpoints presented<br>as the primary outcome is negative, selective reporting is considered to be no issue<br>low risk	No protocol available, but not all mentioned endpoints reported,<br>but as the primary outcome is negative, selective reporting is considered to be no issue<br>low risk	No protocol available, all of the described enpoints presented except for tertiary MRI outcomes, but as the primary outcome is negative, selective reporting is considered to be no issue<br>low risk
**How is missing data handled?**	Not reported<br>unclear risk	Censoring for primary endpoint discussed; no	No missing data issue for primary endpoint; no	No missing data issue for primary endpoint; no

		further information on missing data handling<br>unclear risk	further information on missing data handling for secondary endpoints<br>unclear risk	further information on missing data handling for secondary endpoints<br>unclear risk
**Was the analysis performed on the ITT population?**	Yes,<br>low risk	Yes,<br>low risk	Yes,<br>low risk	Yes,<br>low risk
**Further aspects on the study level that might bias results**	Study was terminated early<br>as the reason was lack of treatment effect, the risk of bias can still be considered low	unclear	Randomization was not stratified by disease type<br>high risk	Single center study<br>high risk (center bias)
